# IL-2 enhanced MHC class I expression in papillary thyroid cancer with Hashimoto's thyroiditis overcomes immune escape* in vitro*

**DOI:** 10.7150/jca.38330

**Published:** 2020-04-27

**Authors:** Jia-Qian Hu, Bo-Wen Lei, Duo Wen, Ben Ma, Ting-Ting Zhang, Zhong-Wu Lu, Wen-Jun Wei, Yu-Long Wang, Yu Wang, Duan-Shu Li, Qing-Hai Ji, Tian Liao

**Affiliations:** Department of Head and Neck Surgery, Fudan University Shanghai Cancer Center; Department of Oncology, Shanghai Medical College, Fudan University, Shanghai 200032, China.

**Keywords:** IL-2, HLA-class I molecule, papillary thyroid cancer, Hashimoto's thyroiditis, CD8+ T cell immunity

## Abstract

The impact of Hashimoto's thyroiditis (HT) on the progression of papillary thyroid cancer (PTC) is still unclear. Interleukin-2 (IL-2) is a growth factor and crucial for HT development. This study aimed at investigating the effect of IL-2 on MHC class I expression in PTC cells and immune activation with experimental treatment for PTC using PTC cell lines. We assessed the expression of IL-2, HLA class I, PD-L1, CD3, CD8 and CD16 molecules in paired PTC tissues and HLA-ABC and PD-L1 expression in IL-2 pre-treated K1, TPC-1 and BCPAP cells by immunohistochemistry, qPCR, flow cytometry and Western blotting. The effect of IL-2 on immunogenicity of PTC cells to stimulate activated human T cells was determined for the percentages of activated CD8+ T cells and their cytokine production as well as PD-1 and PD-L1 expression. Compared with non-tumor tissues, we found that IL-2 expression was up-regulated in PTC tissues, particularly in PTC+HT tissues and correlated positively with HLA-class I, CD3 and CD8 expression in PTC+HT tissues. Conversely, PD-L1 expression decreased in PTC+HT tissues. Treatment with IL-2 significantly up-regulated HLA-class I expression, but down-regulated PD-L1 expression in PTC cells. Co-culture with IL-2-pre-treated PTC cells significantly promoted the proliferation of activated CD8+ T cells and their IL-2 secretion, but decreased their PD-1 expression, accompanied by decreased PD-L1 expression in IL-2-treated PTC cells in vitro. In conclusion, IL-2 up-regulated HLA-class I expression and enhanced anti-tumor T cell immunity during the development of PTC and HT. IL-2 may be a promising immunotherapy for PTC.

## Introduction

The incidence of thyroid cancers (TCs) is increasing substantially during the past several decades, particularly for papillary thyroid cancer (PTC) 1,2. It is notable that PTC is frequently accompanied by Hashimoto's thyroiditis (HT), which is the most common autoimmune thyroid disease 3-5. Extensive studies have shown that PTC with concurrent HT usually behaviors less aggressive 4,6-9, which may be attributed to lymphocytic infiltrates in tumor tissue. Consequently, patients with PTC+HT are more sensitive to immunotherapy. However, it is unclear how local lymphocytic infiltrates modulate the sensitivity of PTC to immunotherapy.

It is well known that immunotherapy is to enhance host's immune cells to identify and destroy tumor cells. In fact, antigen-specific CD8+ T cells are the major players in immunotherapy and crucial for defending against tumor. However, tumor cells, due to low levels of MHC I molecule expression, fail to effectively present antigen determinants to initiate CD8+ T cell immunity, which is one of the mechanisms by which tumors escape immune destruction 10,11. Furthermore, tumor cells also express immune inhibitors, such as PD-L1, and chemokines that recruit regulatory cells 11. Hence, new strategies for immunotherapy should correct immunodeficiency and enhance tumor antigen presentation to induce potent tumor-specific CD8+ T cell immunity and memory.

During the pathogenic process, many immunocompetent cells infiltrate into the lesions to secrete pro-inflammatory cytokines to drive an autoimmune cascade. Interleukin-2 (IL-2) is a multifunctional cytokine, and can promote the proliferation of activated T cells. Recombinant IL-2 has been used for treatment of autoimmune disease and malignancy 12,13. Previous studies have shown that HLA class I molecule expression is usually down-regulated in cancer cells 14-16 and treatment with IFN-γ or selumetinib can enhance HLA molecule expression in PTC cells 15. However, little is known about IL-2 expression and its possible impact on HLA I molecule expression in PTC, particularly in PTC with concurrent HT.

In the present study, we characterized IL-2 and HLA class I expression, and T, CD8+ T cells and NK/monocytes in human PTC, PTC+HT and their adjacent non-tumor tissues. Furthermore, we examined the effect of IL-2 treatment on HLA class I molecule expression in human PTC cells *in vitro* and the impact of IL-2-pre-treated PTC cells on activated CD8+ T cells, their IL-2 production and PD-1 expression as well as PD-L1 expression in PTC cells following co-culture with activated CD8+ T cells *in vitro*. Our findings indicate that IL-2 up-regulates HLA class I molecule expression in PTC cells to enhance CD8+ T cell activation and IL-2 production by down-regulating PD-1/PD-L1 expression *in vitro*.

## Materials and Methods

### Patients and tissue samples

Fresh-frozen thyroid specimens were obtained from 73 patients, who underwent surgical resection of tumors at the Department of Head and Neck Surgery, Fudan University Shanghai Cancer Center between Apr. 2014 and Jan. 2016. All patients with PTC alone or PTC and HT were pathologically confirmed. Their surgical tumors were classified by TNM, according to the 8^th^ AJCC TNM staging system. Their demographic and clinical data (sex, age, tumor size, extra thyroidal invasion, metastasis, multifocality and TNM classification) were collected. All patients signed a written informed consent before the study and this research was approved by the Institutional Review Board of Fudan University Shanghai Cancer Center.

### Immunohistochemistry (IHC)

The expression of IL-2, PD-1, CD3, CD8, CD16 and HLA-ABC in individual PTC and their adjacent non-tumor tissues was examined by IHC. Briefly, the paraffin-embedded tissue sections (4 µm) were deparaffinized, rehydrated and treated with 3% H_2_O_2_in methanol, followed by heat-induced antigen retrieval (0.01mol/L citrate buffer, pH 6.0). After being blocked with 5% bovine serum albumin (BSA), the sections were incubated overnight at 4℃ with antibodies against IL-2 (26156-1-AP, Proteintech), PD-L1(ab140950, Abcam), CD3 (ab16669, Abcam), CD8 (ab17147, Abcam), CD16 (ab203883, Abcam) or HLA Class I ABC (ab70328, Abcam). After being washed, the bound antibodies were detected with horseradish peroxidase (HRP)-conjugated secondary antibodies and visualized with 3,3'-diaminobenzidine (DAB) using a IHC kit (KIHC-1, Proteintech), followed by counterstained with hematoxylin. Images were obtained under an Olympus IX71 microscope (Olympus, Japan) and analyzed by two experienced pathologists in a blinded manner. The immunostaining was scored, according to the percentages of positive cells (0, no staining; 1, ≤10%; 2, 10‑50%; and 3, >50%) and the intensity (0, negative; 1, weak; 2, moderate; and 3, strong), and then were multiplied to get an immunostaining score (IS).

### Cell culture

Human PTC K1, TPC-1 and BCPAP cells and non-tumor thyroid follicular epithelial Nthy-ori 3-1 cells were purchased from University of Colorado Cancer Center Cell Bank (Colorado, USA), Typical Culture Preservation Commission Cell Bank, Chinese Academy of Sciences (Shanghai, China) or Sigma-Aldrich (St. Louis, USA). The cells were cultured in RMPI1640 medium containing 10% fetal bovine serum (FBS, Invitrogen, Carlsbad, CA, USA) at 37℃ with 5% CO_2_.

In some experiments, K1, TPC-1 and BCPAP cells were cultured in 6-well plates and treated in triplicate with 0.1 μg/ml of recombinant human IL-2 (200-02, Peprotech) for 24 h.

### RNA extraction, reverse transcription and quantitative real time PCR (qRT-PCR)

Total RNA was extracted from different groups of cells and tissue samples using TRIzol reagent (Invitrogen) and reversely transcribed into cDNA using the PrimeScript^TM^ RT Reagent Kit (Takara, Dalian, China). The relative levels of IL-2, PD-L1, HLA-A, HLA-B or HLA-C mRNA transcripts to the control β-actin were determined in triplicate by qRT-PCR using SYBR Green Premix Ex Taq^TM^ II (Takara) and specific primers in **Table S1**. The data were normalized to the control and analyzed by 2^-ΔΔCt^**.**

### Preparation of peripheral blood mononuclear cells (PBMCs)

Peripheral blood samples were drawn from healthy volunteers and their PBMCs were isolated by density gradient centrifugation using lymphocyte separation medium (Dakewe Biotech, Shenzhen, China). The isolated PBMCs (2×10^5^cells/well) were cultured in RMPI1640 medium containing 10% FBS (Invitrogen) in U-shaped bottom 96-well plates and treated with 1 μg/mL of anti-CD3 (16-0037-85) and anti-CD28 (16-0289-85, eBioscience) for 72 h. The cells were stained with PE-anti-CD3 (300308), FITC-anti-CD8α (300906) and APC-anti-CD25 (302610, BioLegend) and the activated CD8+ T cells (CD3^+^CD8^+^CD25^+^) were sorted by flow cytometry in a flow cytometer (MoFlo XDP, Beckman Coulter).

### Co-culture of activated CD8+ T cells and PTC cells

The activated CD8+ T cells (effector, 1×10^5^ cells/well)were co-cultured in triplicate with K1, TPC-1 and BCPAP cells (target cells) that had been pretreated with, or without, 0.1 µg/mL of recombinant IL-2 at an E:T ratio of 30 in 24-well plates for 24 h. The supernatants of co-cultured cells were harvested and the cells were collected.

### Enzyme-linked immunosorbent assay (ELISA)

The levels of IL-2 in individual supernatant samples were measured by ELISA using the pre-coated human IL-2 ELISA kit (12-1020-096, Dakewe Biotech), according to the manufacturer's instruction. Briefly, samples and pre-diluted standards were tested in triplicate simultaneously using 3,3'5,5'-tetramethyl benzidinedihydrochloride (TMB) as a substrate and the absorbance of each well was read at 450 nm in a Synergy H4 Hybrid microplate reader (BioTek).

### Flow cytometry

The co-cultured cells were stained with mouse anti-human HLA Class I ABC (ab70328, Abcam) together with FITC-labeled goat anti-mouse secondary antibody (555988, BD PharMingen), PE-anti-CD3 (300308, BioLegend), APC-anti-CD25 (302610, BioLegend) and PE/Cy7-anti-PD-1 (329918, Biolegend). The cells were analyzed by flow cytometry in a Cytomics™ FC 500cytometer (Beckman Coulter) and analyzed using FlowJo software (Tree Star).

### Western blot analysis

Individual groups of cells were lyzed in RIPA lysis buffer containing protease inhibitor and phosphatase inhibitor (Roche, CA, USA). Tissue samples were homogenized and lyzed in T-PER™ Tissue Protein Extraction Reagent (Thermo Scientific), followed by sonication using the Vibra-Cell™ Ultrasonic Liquid Processors (Sonics & Materials). After being centrifuged, the concentrations of total proteins in individual samples were measured by a bicinchoninic acid assay (BCA). The lysate samples (50µg/lane) were resolved by sodium dodecyl sulfate polyacrylamide gel electrophoresis (SDS-PAGE) on 10% gels and transferred onto polyvinylidene difluoride (PVDF) membranes. The membranes were blocked with 5% non-fat dry milk in TBST and probed with anti-HLA class I(1:1000, Abcam), anti-PD-L1 (1:1000, Cell Signaling Technology) and anti-GAPDH (1:5000, Abcam) at 4℃ overnight. After being washed, the membranes were incubated with HRP-conjugated goat anti-rabbit or anti-mouse IgG (1:5000; Jackson ImmunoResearch Laboratories) and visualized with the enhanced chemiluminescent reagents (Thermo Fisher Scientific). The bands were analyzed by Alpha Imager (Alpha Innotech, San Leandro, CA, USA).

### Statistical analysis

All data are shown as mean ± SD or SEM as indicated. Continuous variables were analyzed by one-way analysis of variance (ANOVA) and independent t-tests and categorical variables were analyzed by Pearson's χ2 test using GraphPad Prism 5.01 software (GraphPad Software, Inc.) and SPSS 22.0 (IBM, Armonk, NY, USA). Statistical significance was defined when a *P*-value of <0.05.

## Results

### IL-2 expression is up-regulated in human PTC, particularly in PTC and HT tissues

To determine IL-2 expression, 144 pairs of surgical thyroid tissue specimens were obtained from patients with PTC alone or PTC and HT and their demographic and clinical data are shown in **Table 1.** The percentages of female patients with PTC and HT were significantly higher than those with PTC alone (92.5% vs.71.4%*, P*=0.003), but the frequency of PTC patients with microcarcinoma (< 1 cm) was significantly higher than those with < 1 cm PTC and HT (60.4% vs. 43.4%, *P*=0.048) in this population. There was no significant difference in other measures tested in this population.

**Figure 1:**
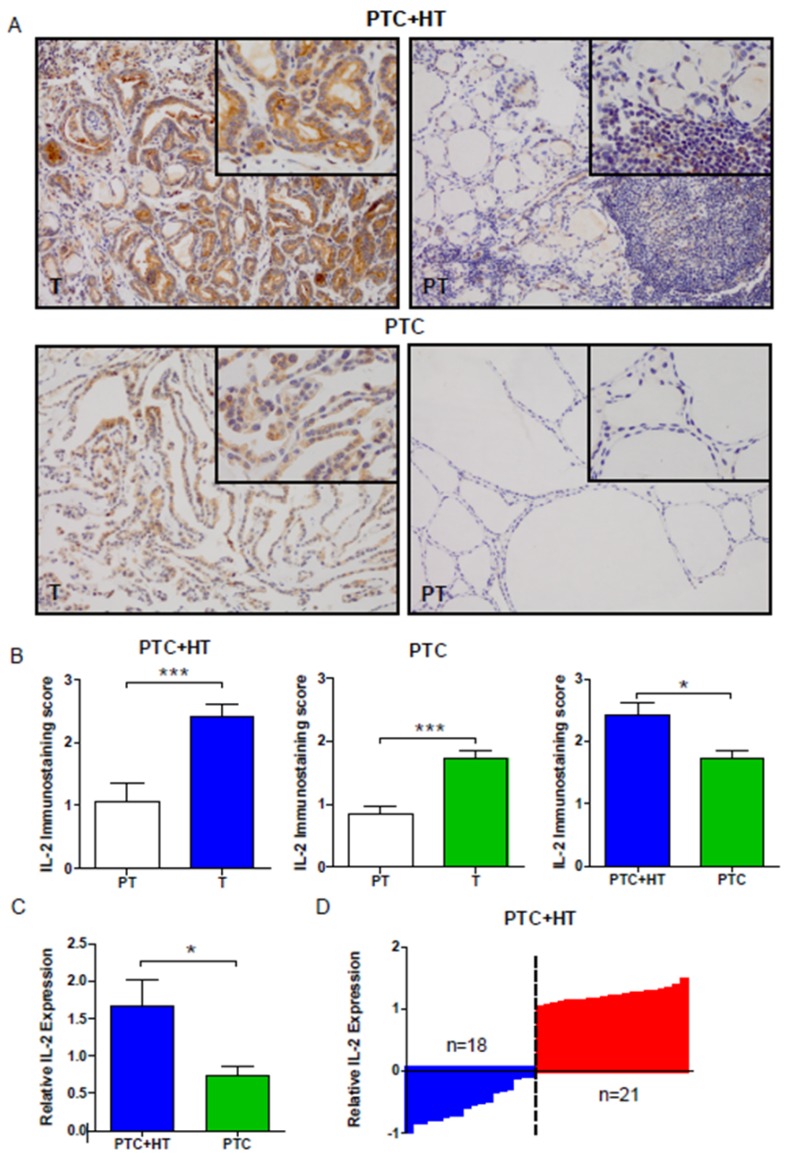
** Up-regulated IL-2 expression in PTC+HT tissues. A**, Immunohistochemistry analysis of IL-2 in 53 PTC+HT, 91 PTC tumor (T) and their adjacent para-non-tumor tissue (PT). **B**, Immunostaining scores of IL-2. **C**, Quantitative RT-PCR analysis of relative IL-2 mRNA transcripts. **D**, The distribution of IL-2 expression in the PTC+HT tissues (n=39). Data are representative images (magnification x 200 with a 400 inserter) or expressed as the mean ± SD of each group. *P<0.05, ***P<0.001

We evaluated the expression of IL-2 in PTC (T) and adjacent para-tumor (PT) tissue samples by IHC. As shown in **Fig. 1A and 1B**, the levels of IL-2 expression were significantly higher in PTC+HT or PTC tumor tissues (T) than in adjacent para-tumor tissue (PT) (*P* < 0.001). IL-2 expression in the PTC+HT tissues was significantly higher than that in PTC tissues (*P* < 0.05). Further qRT-PCR analysis indicated that the relative levels of IL-2 mRNA transcripts in the PTC+HT tissues were significantly higher than that in the PTC tissues (*P* < 0.05, **Fig. 1C**). Stratification analysis of PTC+HT tissues revealed that all higher levels of IL-2 expression were from female patients and the percentages of microcarcinoma in PTC+HT tissues with lower IL-2 expression were significantly higher than those with higher IL-2 expression (*P*=0.042, **Fig. 1D, Table 2**). Hence, IL-2 expression was up-regulated in human PTC, particularly in PTC+HT tissues.

### HLA class I expression increases in PTC+HT, but decreases in PTC tissues

**Figure 2:**
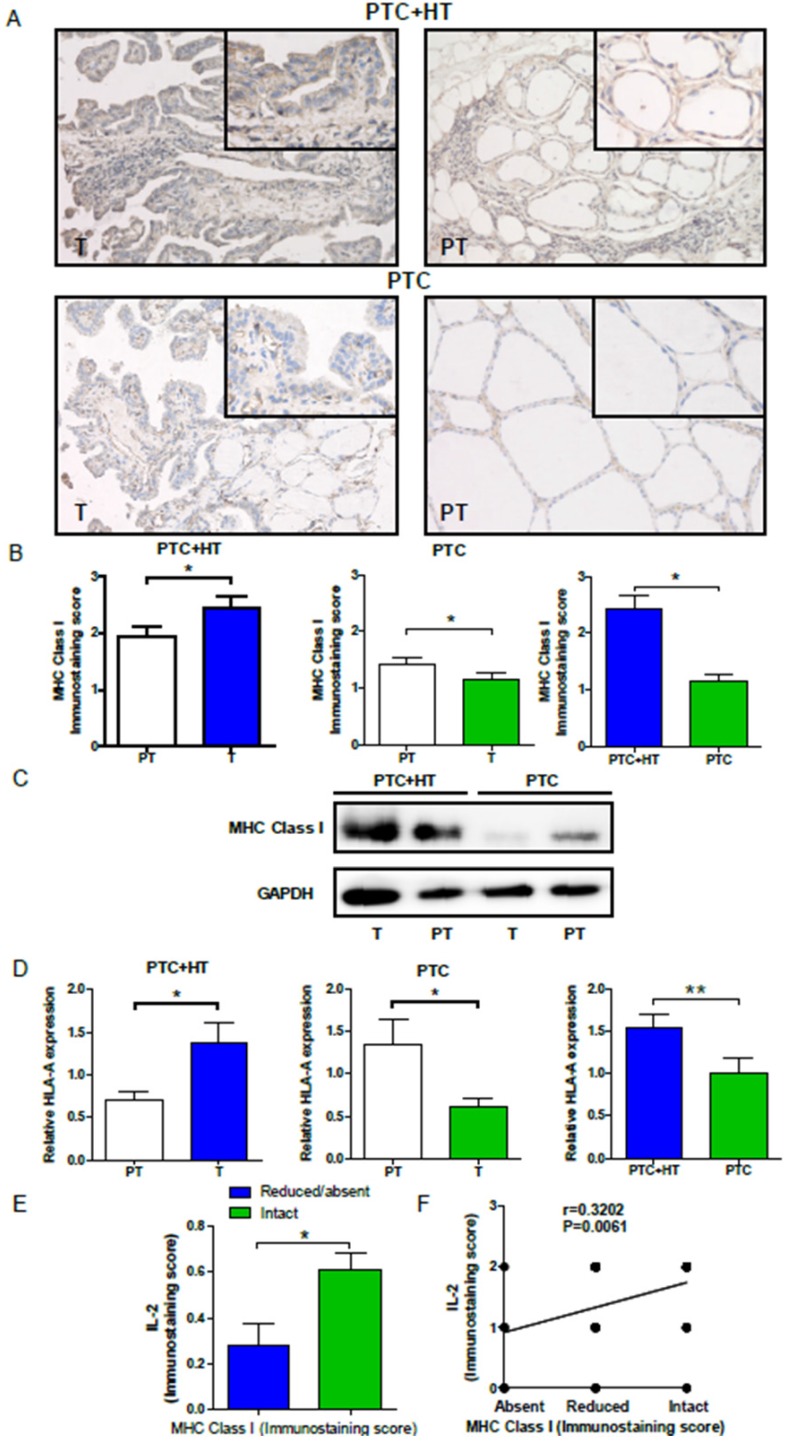
** Up-regulated HLA class I molecule expression in PTC+HT tissues. A**, Immunohistochemistry analysis of HLA class I molecule expression in 53 PTC+HT, 91 PTC tumor (T) and their adjacent para-non-tumor tissue (PT). **B**, Immunostaining scores of HAL class I molecule expression. **C**, Western blot analysis of HLA class I molecule expression. **D**, Quantitative RT-PCR analysis of relative HLA class I molecule mRNA transcripts. **E** and **F**, The relationship between IL-2 and HLA class I molecule expression in PTC+HT tissues. Data are representative images (magnification x 200 with a 400 inserter) or expressed as the mean ± SD of each group. *P<0.05, ***P<0.001.

To understand the potential relationship, HLA class I expression in PTC and PTC+HT tissues was also examined by IHC. As shown in **Fig. 2A and 2B**, HLA class I expression in PTC+HT tumors was significantly higher than that in adjacent para-tumor tissues (*P*<0.05), but it in the PTC tumors was significantly lower than that in adjacent para-tumor tissues(*P*<0.05). Interestingly, HLA class I expression in the PTC+HT tumors was significantly higher than that in the PTC tumors (*P*<0.05). Similar patterns of HLA class I expression and HLA class I-A mRNA transcripts were detected in the different groups of tissue samples by Western blot and qRT-PCR (*P*<0.05, *P*< 0.01, **Fig. 2C and 2D)**. There was no significant difference in the relative levels of HLA class I-B and I-C mRNA transcripts among the different groups of samples (data not shown). Stratification of tumor samples with intact or reduced HLA class I expression indicated that the levels of IL-2 expression in the tumors with intact HLA class I expression were significantly higher than that in those with reduced HLA class I expression (*P*<0.05, **Fig. 2E).** Moreover, the levels of IL-2 expression were positively correlated with HLA class I expression in the tumor tissues in this population** (***r=*0.3203, *P*=0.006, **Fig. 2F).** Such data indicated that IL-2 expression was correlated with HLA class I expression in human PTC and PTC+HT tissues and suggest that IL-2 may up-regulate HLA class I expression in human PTC and PTC+HT tissues.

### Immune cells infiltrate in PTC and PTC+ HT tissues

**Figure 3:**
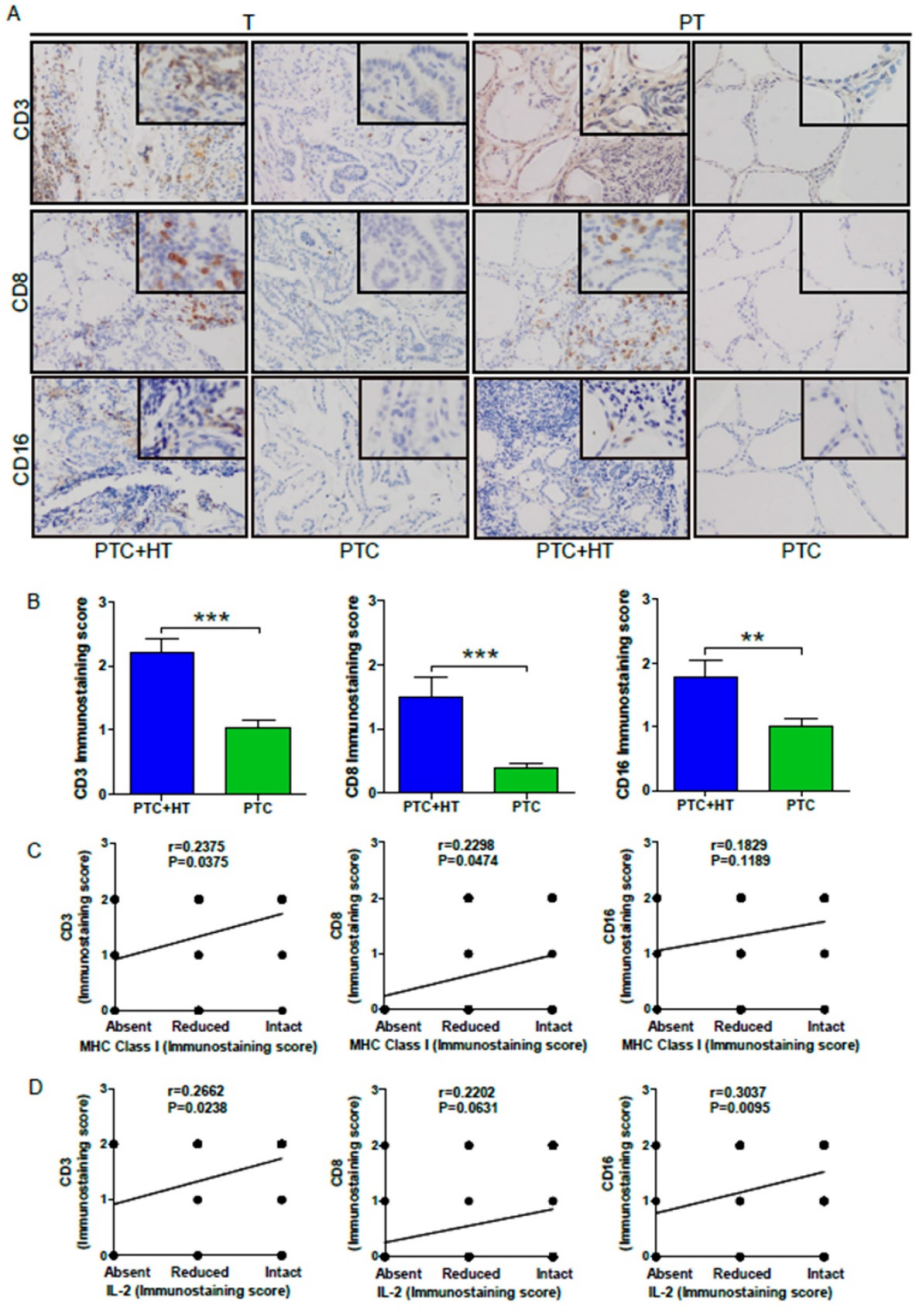
** UP-regulated CD3, CD8 and CD16 expression in PTC+HT tissues. A**, IHC analysis of CD3, CD8, and CD16 expression in 53 PTC+HT, 91 PTC tumor (T) and their adjacent para-non-tumor tissue (PT).** B**, Immunostaining scores ofCD3, CD8, and CD16 expression in tumor tissues. **C**, The correlation between the immunostaining scores of HLA class I molecule and CD3, CD8 and CD16 expression. **D**, The correlation between the immunostaining scores of IL-2 and CD3, CD8 and CD16 expression. Data are representative images (magnification x 200 with a 400 inserter) or expressed as the mean ± SD of each group. *P<0.05, **P<0.01, ***P<0.001.

Because elevated MHC class I molecules may recruit more leukocyte infiltrates in the tumor tissues, we next examined CD3, CD8 and CD16 expression to reflect T, CD8+ T and NK/monocytes in the PTC and PTC+HT tissues by IHC. As shown in **Figure 3A,**CD3, CD8 and CD16 expression were detected in PTC+HT tumors and their adjacent para-tumor tissues, but little in the PTC tissues and their adjacent non-tumor tissues, indicating that T and CD8+ T cells as well as monocytes infiltrated predominantly in the PTC+HT and their adjacent non-tumor tissues. Semi-quantitative analyses indicated that the intensity of anti-CD3, anti-CD8 and anti-CD16 staining in the PTC+HT tissues was significantly stronger than that in the PTC tissues (*P*<0.001, *P*<0.01, **Fig. 3B**). Stratification analyses revealed that the levels of anti-CD3 (*r=*0.2375, *P*=0.0375) and anti-CD8 (*r=*0.2298, *P*=0.0474), but not anti-CD16 (*r=*0.1829, *P*=0.1189) staining in the PTC+HT tissues were positively correlated with HLA class I expression (**Fig. 3C**), and the levels of IL-2 expression were positively correlated with anti-CD3 (*r=*0.2662, *P*=0.0238) and anti-CD16 (*r=*0.3037, *P*=0.0095), but not significantly with anti-CD8 staining in the PTC+HT tissues (*r=*0.2202, *P*=0.0631, **Fig. 3D**). Therefore, up-regulated IL-2 and HLA class I expression were associated with more immunocompetent cell infiltration in PTC+HT tissues.

### IL-2 up-regulates HLA class I molecule expression in PTC cells

**Figure 4:**
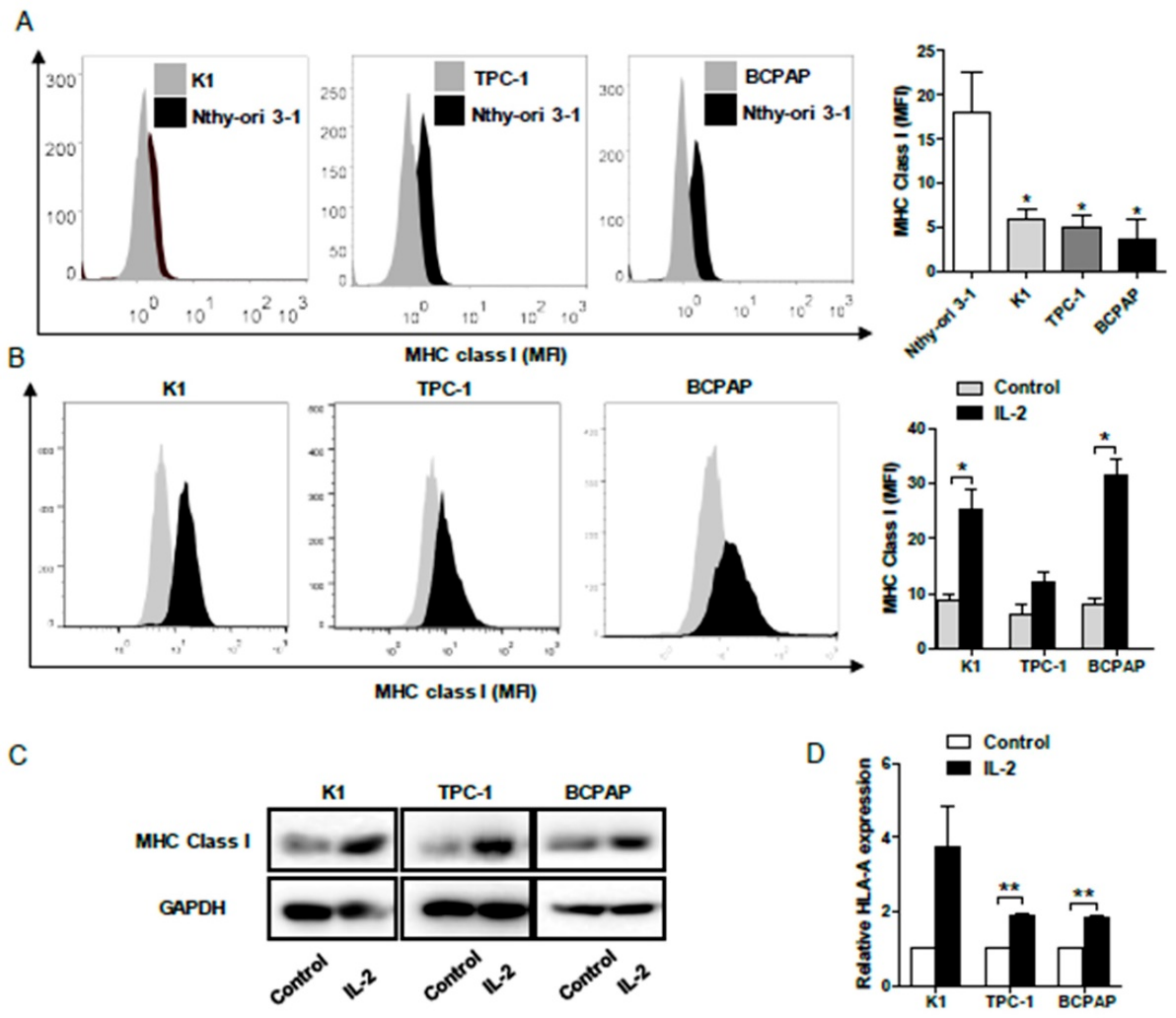
** IL-2 up-regulates HLA class I molecule expression in PTC cells. A** and **B**, Flow cytometry analysis of HLA class I molecule expression in K1, TPC-1 and BCPAP and non-tumor Nthy-ori 3-1 cells following treatment with, or without, IL-2. **C** and **D**, Western blot and qRT-PCR analyses of HLA class I molecule expression in PTC cells that had been treated with, or without, IL-2. Data are representative histograms or expressed as the mean ± SD of each group. *P<0.05, **P<0.01, ***P<0.001.

To determine the impact of IL-2 on HLA class I molecule expression, human PTC K1, TPC-1, BCPAP and non-tumor Nthy-ori 3-1 cells were treated with, or without, recombinant IL-2 (0.1μg/mL) for 24h and the levels of HLA class I expression was characterized by flow cytometry. First, the levels of HLA class I molecule expression in PTC cells were significantly lower than that in non-tumor Nthy-ori 3-1 cells (*P*< 0.05, **Fig. 4A**). Following treatment with IL-2, the levels of HLA class I molecule expression increased significantly in K1 and BCPAP cells, but not in TPC-1 cells (*P*<0.05 for all, **Fig. 4B).** Further Western blot and qRT-PCR indicated that treatment with IL-2 significantly increased HLA-class I molecule expression in all PTC cells (*P*<0.01 for all, **Fig. 4C and 4D**). Such data clearly demonstrated that IL-2 up-regulated HLA class I molecule expression in PTC cells *in vitro*.

### IL-2 up-regulated HLA class I molecule expression promotes T cell activation and IL-2 production, but suppresses PD-1/PD-L1 expression *in vitro*

**Figure 5:**
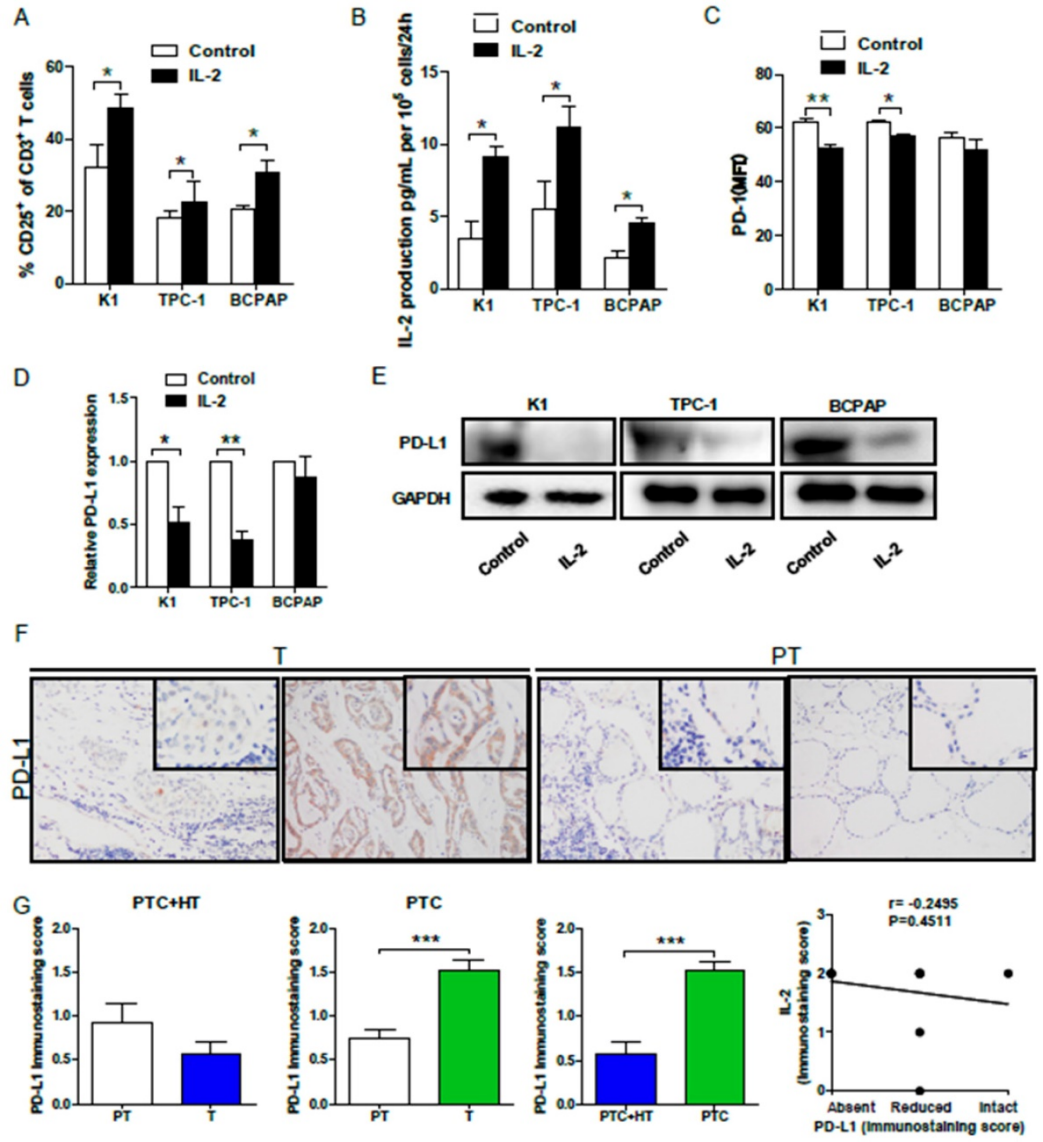
** IL-2 promotes the proliferation, cytokine production, but reduces PD-1 expression in effector CD8+ T cells and PD-L1 in PTC cells.** Effector CD3^+^CD8^+^CD25^+^T cells were co-cultured with the indicated PTC cells that had been pre-treated with, or without, IL-2 (0.1 μg/μL) for 24 h and the frequency of CD3^+^CD8^+^CD25^+^T cells and the levels of PD-1 expression were determined by flow cytometry. The levels of IL-2 in the supernatants were analyzed by ELISA and the relative levels of PD-L1 expression in PTC cells were analyzed by qRT-PCR and Western blot. Furthermore, the PD-L1 expression in PTC and PTC+HT tissues and their para-non-tumor tissues was analyzed by IHC. **A**, The frequency of CD3^+^CD8^+^CD25^+^T cells. **B**, The levels of IL-2. **C**, PD-1 expression in effector CD8^+^T cells. **D** and **E**, PD-L1 expression in PTC cells. **F**, IHC analysis of PD-L1 expression in tissues. **G**, Immunostaining scores of PD-L1 expression. **H**, The correlation between the immunostaining scores of IL-2 and PD-L1. Datas are representative images (magnification x 200 with a 400 inserter) or expressed as the mean ± SD of each group. *P<0.05, **P<0.01, ***P<0.001.

Finally, we examined whether IL-2 up-regulated HLA class I molecule expression could modulate effector T cell activation *in vitro*. Allogeneic activated CD3^+^CD8^+^CD25^+^T cells (effector cells, E) were co-cultured with 30:1 K1, TPC-1 and BCPAP cells (target cells, T) that had been pre-treated with, or without, IL-2. We found that co-culture with IL-2-pre-treated PTC cells significantly increased the percentages of activated CD25+ T cells (*P*<0.05 for all, **Fig. 5A**) and their IL-2 production (*P*<0.05 for all, **Fig. 5B**). Interestingly, flow cytometry analysis indicated that co-culture with IL-2-pre-treated K1 or TPC-1, but not BCPAP cells, significantly decreased PD-1 expression in effector CD8+ T cells (*P*<0.01, *P*<0.05, **Fig. 5C**). Furthermore, co-culture with effector CD8+ T cells significantly reduced the levels of PD-L1 mRNA transcripts in IL-2-pretreated K1 and TPC-1, but not BCPAP, cells (*P*<0.05, *P*<0.01, **Fig. 5D**). Western blot analysis exhibited that co-culture with effector CD8+ T cells also decreased PD-L1 expression in IL-2-pretreated PTC cells (**Fig. 5E**). Moreover, IHC revealed thatPD-L1 expression in PTC+HT tumor tissues were slightly lower than their adjacent non-tumor tissues, and the levels of PD-L1 expression in PTC tumor tissues were significantly higher than that in their adjacent non-tumor tissues and the PTC+HT tumor tissues (*P*<0.001, **Fig. 5F and 5G**). However, there was no significant correlation between PD-L1 and IL-2 expression in PTC and PTC+HT tissues (**Fig. 5H**). Therefore, following co-culture, IL-2-upregulated HLA class I molecule expression enhanced CD8+ T cell responses and IL-2 production, but decreased their PD-1 expression in T cells and PD-L1 expression in PTC cells.

## Discussion

PTC is usually accompanied by HT, and is less aggressive than PTC alone 4-8. A previous study has shown that HT may be a protective factor for the progression of PTC 7. However, the precise mechanisms underlying the progression of PTC remain unclear 4,9. Given that many immune T cells can infiltrate in the inflammatory lesions and they can secrete IL-2 17,18, we examined IL-2 and MHC class I expression in PTC and PTC+HT tissues. We found that IL-2 expression was up-regulated in PTC and PTC+HT tissues, particularly for PTC+HT tissues, extending previous observation that elevated serum IL-2 in HT patients 19,20. Interestingly, many microcarcinoma PTC+HT tissues displayed lower levels of IL-2 expression in this population. Given that microcarcinoma PTC has less recurrent chance 21, the lower levels of IL-2 expression may reflect less pro-inflammatory infiltrates in the tumor lesions. Thus, IL-2 expression may be valuable for diagnosis of PTC and PTC+HT.

MHC class I expression is crucial for antitumor CD8+ T cell immunity. Loss of MHC class I expression is one of the frequent characters in tumor tissues and may be associated with immune escape of malignant tumors, including PTC 14-16,22. In this study, we found that MHC class I expression significantly decreased in PTC tissues, but increased in PTC+HT tissues. Moreover, HLA class I molecule expression was positively correlated with IL-2 in PTC+HT tissues. In addition, HLA class I molecule or IL-2 expression was positively correlated with levels of CD3 or CD8 expression in PTC+HT tissues. Furthermore, treatment with IL-2 significantly up-regulated HLA class I molecule expression in PTC cells. More importantly, co-culture with IL-2-treated PTC cells significantly increased the proliferation and cytokine production of effector CD8+ T cells *in vitro*. Collectively, our data suggest that T cell infiltrates may through secreting IL-2, up-regulate HLA class I molecule expression to enhance the immunogenicity of PTC and promote T cell immunity in PTC tissue. Such novel findings may provide a reasonable explanation why PTC concurrently with HT usually has a better prognosis in the clinic 6-8.

It is well known that PD-L1/PD-1 co-stimulation signaling negatively regulates T cell immunity and promotes the escape of tumors from immunosurveillance 23-25. In this study, we found that IL-2-treated PTC cells significantly decreased PD-1 expression in effector CD8+ T cells and PD-L1 expression in PTC cells, consistent with reduced levels of PD-L1 in PTC+HT tissues. Our data extended previous observation that IL-2 can overcome PD-L1-mediated inhibition in murine CD8^+^T cells 26. The reduced PD-1 and PD-L1 expression indirectly evidenced stronger antitumor T cell immunity. Given that positive PD-L1 expression in PTC tissues was significantly associated with worse recurrence-free survival^27^, the IL-2-reducedPD-L1 expression in PTC cells, together with decreased PD-L1 expression in PTC+HT tissues, suggests that IL-2 may enhance antitumor T cell immunity to limit the progression of PTC. Accordingly, our findings may provide new insights into regulating the pathogenic process of PTC.

This study had limitations, including the lack of relevant survival data in this population due to insufficient data, smaller sample size that might cause a biased, analyzing only IL-2 although many cytokines and chemokines in the HT environment as well as no direct therapeutic evaluation of IL-2 *in vivo* due to lack of an optimal animal model. Thus, further investigation of a bigger population with multiple cytokines for a longer period is warranted.

In conclusion, our data indicated that IL-2 expression was up-regulated and positively correlated with HLA class I molecule expression in PTC+HT tissues. IL-2 also significantly up-regulated HLA class I molecule expression in PTC cells. Co-culture with IL-2-treated PTC cells significantly increased the proliferation and cytokine production, but decreased PD-1 expression in activated CD8+ T cells and reduced PD-L1 expression in PTC cells. Accordingly, IL-2 up-regulated HLA class I molecule expression and increased the immunogenicity of PTC cells to enhance T cell immunity, limiting the progression of PTC. Therefore, our findings may provide new explanation why patients with PTC concurrently with HT have a better prognosis in clinic and suggest that IL-2 may be a valuable immunotherapy for PTC.

## Supplementary Material

Supplementary table.

## Figures and Tables

**Table 1:** The demographic and clinical characteristics of patients (n=144)

Clinicopathologic parameters	PTC(n=91)	PTC+HT(n=53)	*p*-value
	N(%)	N(%)	
**Gender**			0.003*
Male	26 (28.6)	4(7.5)	
Female	65 (71.4)	49(92.5)	
**Age (years)**			
Mean	42.7 ± 12.3	39.9 ± 13.0	0.195
< 55	75(82.4)	46(86.8)	0.489
≥ 55	16(17.6)	7(13.2)	
**Tumor size (cm)**			
Mean	1.1 ± 0.7	1.4 ± 1.0	0.065
≤ 1	55(60.4)	23(43.4)	0.048*
> 1	36(39.6)	30(56.6)	
**Multifocal lesions**			0.083
Positive	25(27.5)	22(42.5)	
Negative	66(72.5)	31(58.5)	
**Extrathyroidal invasion**			0.215
Positive	9(9.9)	9(17.0)	
Negative	82(90.1)	44(83.0)	
**Lymph node metastasis**			0.489
Positive	46(50.5)	30(56.6)	
Negative	45(49.5)	23(43.4)	
**TNM stage 8^th^**			0.278
I, II	90(98.9)	51(96.2)	
III, IV	1(1.1)	2(3.8)	

^*^P< 0.05

**Table 2:** The demographic and clinical characteristics of PTC+HT patients (n=39)

Clinicopathologic parameters	IL-2^Low^	IL-2^High^	*p*-value
	N(%)	N(%)	
**Gender**			0.052
Male	3(16.7)	0(0)	
Female	15(83.3)	21(100)	
**Age (years)**			
Mean	44.5 ± 13.0	39.2 ± 14.0	0.235
< 55	15(83.3)	18(85.7)	0.837
≥ 55	3(16.7)	3(14.3)	
**Tumor size (cm)**			
Mean	1.2± 1.1	1.3 ± 0.6	0.915
≤ 1	10(55.6)	5(23.8)	0.042*
> 1	8(44.4)	16(76.2)	
**Multifocal lesions**			0.802
Positive	7(38.9)	9(42.9)	
Negative	11(61.1)	12(57.1)	
**Extrathyroidal invasion**			0.298
Positive	5(27.8)	3(14.3)	
Negative	13(72.2)	18(85.7)	
**Lymph node metastasis**			0.882
Positive	9(50.0)	11(52.4)	
Negative	9(50.0)	10(47.6)	
**TNM stage 8^th^**			0.117
I, II	16(88.9)	21(100)	
III, IV	2(11.1)	0(0)	

^*^P< 0.05

## References

[1] La VC, Malvezzi M, Bosetti C, Garavello W, Bertuccio P, Levi F (2015). Thyroid cancer mortality and incidence: a global overview. International Journal of Cancer.

[2] Wiltshire JJ, Drake TM, Uttley L, Balasubramanian SP (2016). Systematic Review of Trends in the Incidence Rates of Thyroid Cancer. Thyroid.

[3] Dailey ME, Lindsay S, Skahen R (1955). Relation of thyroid neoplasms to Hashimoto disease of the thyroid gland. AMA archives of surgery.

[4] Caturegli P, De Remigis A, Chuang K, Dembele M, Iwama A, Iwama S (2013). Hashimoto's thyroiditis: celebrating the centennial through the lens of the Johns Hopkins hospital surgical pathology records. Thyroid.

[5] Konturek A, Barczyński M, Wierzchowski W, Stopa M, Nowak W (2013). Coexistence of papillary thyroid cancer with Hashimoto thyroiditis. Langenbecks Arch Surg.

[6] Lun Y, Wu X, Xia Q, Han Y, Zhang X, Liu Z (2013). Hashimoto's thyroiditis as a risk factor of papillary thyroid cancer may improve cancer prognosis. Otolaryngology-Head and Neck Surgery.

[7] Zhang L, Li H, Ji Q-h, Zhu Y-x, Wang Z-y, Wang Y (2012). The clinical features of papillary thyroid cancer in Hashimoto's thyroiditis patients from an area with a high prevalence of Hashimoto's disease. BMC cancer.

[8] Alcântara-Jones DMd, Alcântara-Nunes TFd, Rocha BdO, Oliveira RDd, Santana ACP, Alcântara FTd (2015). Is there any association between Hashimoto's thyroiditis and thyroid cancer? A retrospective data analysis. Radiologia brasileira.

[9] Nam HY, Lee H, Park G (2016). Impact of co-existent thyroiditis on clinical outcome in papillary thyroid carcinoma with high preoperative serum antithyroglobulin antibody: a retrospective cohort study. Clinical Otolaryngology.

[10] Lechner MG, Russell SM, Bass RS, Epstein AL (2011). Chemokines, costimulatory molecules and fusion proteins for the immunotherapy of solid tumors. Immunotherapy.

[11] Stewart TJ, Abrams SI (2008). How tumours escape mass destruction. Oncogene.

[12] Hughes T, Klairmont M, Broucek J, Iodice G, Basu S, Kaufman HL (2015). The prognostic significance of stable disease following high-dose interleukin-2 (IL-2) treatment in patients with metastatic melanoma and renal cell carcinoma. Cancer Immunology Immunotherapy.

[13] Brivio F, Lissoni P, Alderi G, Barni S, Lavorato F, Fumagalli L (2016). Preoperative interleukin-2 subcutaneous immunotherapy may prolong the survival time in advanced colorectal cancer patients. Oncology.

[14] Paulson KG, Tegeder A, Willmes C, Iyer JG, Afanasiev OK, Schrama D (2014). Downregulation of MHC-I expression is prevalent but reversible in Merkel cell carcinoma. Cancer immunology research. 2014: canimm. 0005.

[15] Angell TE, Lechner MG, Jang JK, LoPresti JS, Epstein AL (2014). MHC class I loss is a frequent mechanism of immune escape in papillary thyroid cancer that is reversed by interferon and selumetinib treatment in vitro. Clinical Cancer Research.

[16] Siddle HV, Kreiss A, Tovar C, Yuen CK, Cheng Y, Belov K (2013). Reversible epigenetic down-regulation of MHC molecules by devil facial tumour disease illustrates immune escape by a contagious cancer. Pnas.

[17] Calcinotto A, Grioni M, Jachetti E, Curnis F, Mondino A, Parmiani G (2012). Targeting TNF-Î± to neoangiogenic vessels enhances lymphocyte infiltration in tumors and increases the therapeutic potential of immunotherapy. Journal of Immunology.

[18] Shrimali RK, Yu Z, Theoret MR, Chinnasamy D, Restifo NP, Rosenberg SA (2010). Antiangiogenic Agents Can Increase Lymphocyte Infiltration into Tumor and Enhance the Effectiveness of Adoptive Immunotherapy of Cancer. Cancer Research.

[19] Figueroa-Vega N, Alfonso-Perez M, Benedicto I, Sanchez-Madrid F, Gonzalez-Amaro R, Marazuela M (2010). Increased circulating pro-inflammatory cytokines and Th17 lymphocytes in Hashimoto's thyroiditis. The Journal of Clinical Endocrinology & Metabolism.

[20] Yaylali GF, Guleryuz B, Akin F, Turgut S, Topsakal S, Ata MT (2015). IL-2, IL-4, IL-5, IFN-[gamma] and TNF-[alpha] levels in Turkish patients with Hashimoto's thyroiditis. Endocrine.

[21] Chereau N, Trésallet C, Noullet S, Godiris-Petit G, Tissier F, Leenhardt L (2016). Does the T1 subdivision correlate with the risk of recurrence of papillary thyroid cancer?. Langenbecks Archives of Surgery.

[22] Zhou F, Chen J, Zhao KN (2013). Human papillomavirus 16-encoded E7 protein inhibits IFN-γ-mediated MHC class I antigen presentation and CTL-induced lysis by blocking IRF-1 expression in mouse keratinocytes. Journal of General Virology.

[23] Akbay EA, Koyama S, Carretero J, Altabef A, Tchaicha JH, Christensen CL (2013). Activation of the PD-1 pathway contributes to immune escape in EGFR-driven lung tumors. Cancer discovery.

[24] French JD, Kotnis GR, Said S, Raeburn CD, McIntyre Jr RC, Klopper JP (2012). Programmed death-1+ T cells and regulatory T cells are enriched in tumor-involved lymph nodes and associated with aggressive features in papillary thyroid cancer. The Journal of Clinical Endocrinology & Metabolism.

[25] Imam S, Paparodis R, Sharma D, Jaume JC (2014). Lymphocytic profiling in thyroid cancer provides clues for failure of tumor immunity. Endocrine-related cancer.

[26] Carter LL, Fouser LA, Jussif J, Fitz L, Deng B, Wood CR (2002). PD-1:PD-L inhibitory pathway affects both CD4(+) and CD8(+) T cells and is overcome by IL-2. European Journal of Immunology.

[27] Shi RL, Qu N, Luo TX, Xiang J, Liao T, Sun GH (2017). Programmed Death-Ligand 1 Expression in Papillary Thyroid Cancer and Its Correlation with Clinicopathologic Factors and Recurrence. Thyroid.

