# Supplementary material for: IL-2 enhanced MHC class I expression in papillary thyroid cancer with Hashimoto's thyroiditis overcomes immune escape in vitro
Source: J Cancer. 2020 Apr 27;11(14):4250–60. doi: 10.7150/jca.38330 (PMC7196247; doi:10.7150/jca.38330)
Supplement: Supplementary file 1 — Supplementary table. [file jcav11p4250s1.pdf]

Supplementary Table 1. The sequences of primers.

Gene	Forward (5'-3')	Reverse (5'-3')
IL-2	AGAACTCAAACCTCTGGAGGAAG	GCTGTCTCATCAGCATATTCACAC
PD-L1	CCATACAGCTGAATTGGTCATC	CAGAATTACCAAGTGAGTCCTTTCA
HLA-A	GTGGCCTCATGGTCAGAGAT	GCAGTTGAGAGCCTACCTGG
HLA-B	GTGATCTCCGCAGGGTAGAA	TCCGCAGATACCTGGAGAAC
HLA-C	TGATCTCCGCAGGGTAGAAG	CAGATACCTGGAGAACGGGA
β-Actin	CACCATTGGCAATGAGCGGTTC	AGGTCTTTGCGGATGTCCACGT
